# Unexpected loss of TAS1R1–TAS1R3 umami taste receptor function in carnivorous Lyncodontini mustelids

**DOI:** 10.1093/chemse/bjaf045

**Published:** 2025-10-23

**Authors:** Mieczyslaw Wolsan, Jun J Sato

**Affiliations:** Museum and Institute of Zoology, Polish Academy of Sciences, Twarda 51/55, 00-818 Warszawa, Poland; Department of Biological Science, Fukuyama University, Higashimura-cho, Aza, Sanzo, 985-1, Fukuyama 729-0292, Japan

**Keywords:** feeding specialization, *Galictis*, gustation, loss of function, *Lyncodon*, nonadaptive convergence

## Abstract

*Lyncodon patagonicus* (Patagonian weasel), *Galictis cuja* (lesser grison), and *Galictis vittata* (greater grison) are the only extant species of Lyncodontini, a relatively poorly known Neotropical tribe of the mustelid subfamily Ictonychinae within the mammalian order Carnivora. Here, we report molecular evidence indicating that the TAS1R1–TAS1R3 umami (savory) taste receptor lost its function in the Lyncodontini's stem lineage (∼3 to 9.5 million years ago) and is therefore nonfunctional in all crown Lyncodontini. This finding is unexpected and intriguing because all extant Lyncodontini apparently need this receptor (they are terrestrial carnivores with diets high in umami-eliciting compounds, including purine 5′-monophosphate ribonucleotides, the main agonists of TAS1R1–TAS1R3 in carnivorans). We argue that the common ancestor of extant Lyncodontini that first lost TAS1R1–TAS1R3 function was semiaquatic and predated mainly on fish and/or aquatic invertebrates (tissues of living or recently dead fish and aquatic invertebrates are low in purine 5′-monophosphate ribonucleotides). This hypothesis is consistent with the idea that loss of taste receptor function is caused by feeding specializations that restrict access to the compounds that a particular receptor detects. Our hypothesis effectively suggests a prolonged semiaquatic episode in the evolutionary history of the Lyncodontini's stem lineage because loss of TAS1R1–TAS1R3 function is achieved by a stochastic process continuing over evolutionary time. Whether the extant Lyncodontini evolved a mechanism to compensate for the loss of TAS1R1–TAS1R3 function is currently unknown and requires further research.

## 1. Introduction

The vertebrate oral cavity is equipped with chemosensory receptors that enable sensation of a variety of tastes or taste qualities, of which sweet, umami (savory), bitter, salty, and sour are universally accepted as basic. While several receptors have been reported to recognize umami-eliciting compounds, including taste mGluR4 (Chaudhari et al. 2000), TAS1R1–TAS1R3 (Li et al. 2002; Nelson et al. 2002), and taste mGluR1 (San Gabriel et al. 2005), the TAS1R1–TAS1R3 heterodimer is widely regarded as the primary umami taste receptor (Munger and Meyerhof 2015; Roura and Foster 2018; Töle et al. 2019). The TAS1R3 protein also heterodimerizes with the TAS1R2 protein to act as the sweet taste receptor (Nelson et al. 2001). The heterodimerization is vital for the function of both receptors, with the result that a loss of integrity of one of the component TAS1Rs causes the loss or severe reduction of function of either receptor (Nelson et al. 2001, 2002; Li et al. 2002; Zhao et al. 2003; Chaudhari et al. 2009).

The taste of umami is considered to be typically evoked by proteinogenic amino acids (Ikeda 1909; Nelson et al. 2002) and potentiated in the presence of purine 5′-monophosphate ribonucleotides (Kuninaka 1960; Yoshii et al. 1986). However, it has been demonstrated that in various mammals, including carnivorans, TAS1R1–TAS1R3 exhibits higher sensitivity to purine 5′-monophosphate ribonucleotides than to amino acids (Toda et al. 2021a; McGrane et al. 2023). Purine 5′-monophosphate ribonucleotides are abundant in tissues of tetrapods (van Waarde 1988; Yamaguchi and Ninomiya 2000; Kurihara 2009; Kaneko et al. 2014). Therefore, carnivorans that regularly feed on tetrapods are expected to possess a functional TAS1R1–TAS1R3.

Here, we report molecular evidence for the loss of TAS1R1–TAS1R3 function in Lyncodontini, a relatively poorly known Neotropical tribe of the mustelid subfamily Ictonychinae within the mammalian order Carnivora (note that we refer the name Lyncodontini to a crown clade). This finding is unexpected and intriguing because the diets of the extant species of this tribe are apparently rich in purine 5′-monophosphate ribonucleotides. Specifically, *Lyncodon patagonicus* (Patagonian weasel) preys on fossorial rodents and birds (Prevosti et al. 2009), and *Galictis cuja* (lesser grison) and *Galictis vittata* (greater grison) feed almost exclusively on small- to medium-sized tetrapods (Yensen and Tarifa 2003a, 2003b). In this article, we will attempt to find an explanation for this puzzle.

## 2. Materials and methods

To evaluate whether or not TAS1R1–TAS1R3 is functional in Lyncodontini, we examined the integrity of the genes that encode this receptor's proteins. Specifically, we analyzed the full-length coding sequences of the *TAS1R1* and *TAS1R3* genes of *L. patagonicus* and *G. cuja* in search for inactivating mutations that convert functional genes into nonfunctional pseudogenes.

The DNA sequence data for *TAS1R1* and *TAS1R3* of *L. patagonicus* and *TAS1R1* of *G. cuja* were generated with the protocol described in Wolsan and Sato (2022) using the samples listed in Supplementary Table S1 and primers listed in Supplementary Tables S2–S4. These sequence data were manually aligned to the *TAS1R3* sequence data of *G. cuja* and the *TAS1R1* and *TAS1R3* sequence data of *Ictonyx striatus* (striped polecat) and *Vormela peregusna* (marbled polecat), all generated by us previously (Wolsan and Sato 2022; DDBJ/ENA/GenBank accession numbers LC654496, LC727912, LC727945, LC727953, and LC727975) and the *TAS1R1* and *TAS1R3* sequence data of *Canis familiaris* (domestic dog) retrieved from DDBJ/ENA/GenBank (accession number GCA_000002285.2).

## 3. Results

**Fig. 1.**
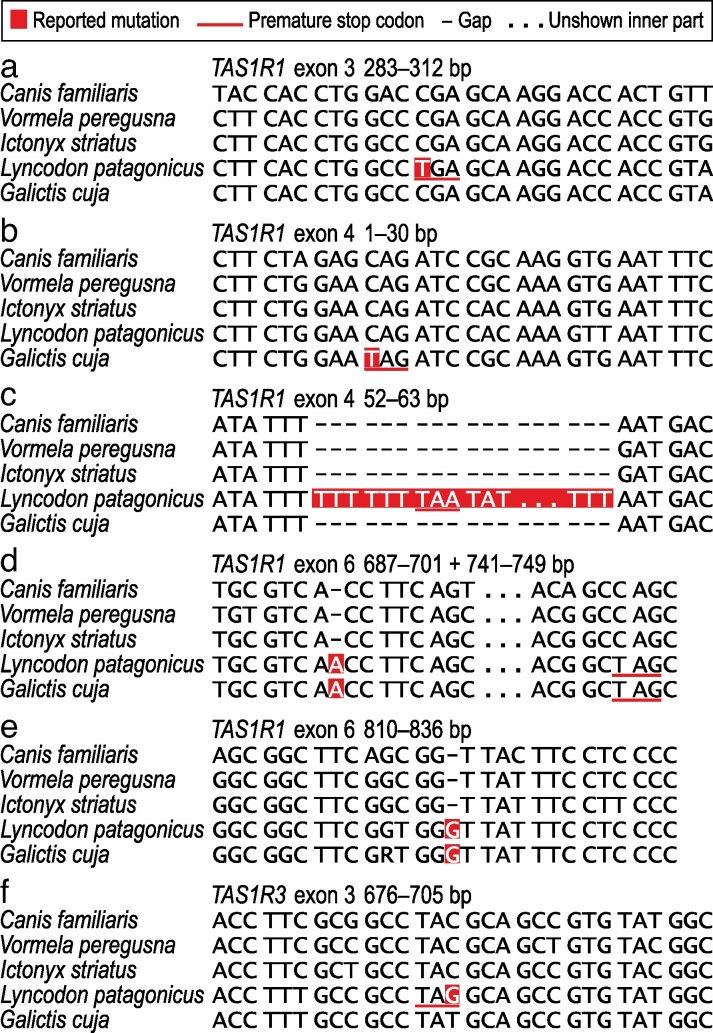
DNA sequence data alignments showing *TAS1R1* and *TAS1R3* inactivating mutations in *L. patagonicus* and *G. cuja*. a) Homozygous substitution of C to T at 295 bp in *TAS1R1* exon 3 of *L. patagonicus* introducing a premature homozygous stop codon (TGA, 295 to 297 bp); b) homozygous substitution of C to T at 10 bp in *TAS1R1* exon 4 of *G. cuja* introducing a premature homozygous stop codon (TAG, 10 to 12 bp); c) >210-bp insertion (shown are only the 5′-terminal 12 bp and 3′-terminal 3 bp) between 57 and 58 bp in *TAS1R1* exon 4 of *L. patagonicus* introducing a premature homozygous stop codon (TAA); d) 1-bp insertion between 693 and 694 bp in *TAS1R1* exon 6 of *L. patagonicus* and *G. cuja* introducing a premature homozygous stop codon (TAG) at 746 to 748 bp; e) 1-bp insertion between 823 and 824 bp in *TAS1R1* exon 6 of *L. patagonicus* and *G. cuja* not introducing a premature stop codon; f) homozygous substitution of C or T to G at 690 bp in *TAS1R3* exon 3 of *L. patagonicus* introducing a premature homozygous stop codon (TAG, 688 to 690 bp). Numbering of base pair positions refers to the aligned sequences of *C. familiaris* and 2 non-Lyncodontini ictonychines (*V. peregusna* and *I. striatus*), starts from the 5′ end of each exon separately, and is from left to right. Codons in the correct open reading frame are separated by spaces.

There are 2 nonsense and 2 frameshift mutations in *TAS1R1* of *L. patagonicus* (Fig. 1a and c–e), 1 nonsense and 2 frameshift mutations in *TAS1R1* of *G. cuja* (Fig. 1b, d, and e), and 1 nonsense mutation in *TAS1R3* of *L. patagonicus* (Fig. 1f). Whereas the nonsense mutations are species-specific (Fig. 1a–c, f), the frameshift mutations are identical between both species (Fig. 1d and e).

One frameshift and all nonsense mutations are accompanied by a premature stop codon, which effectively truncates the coding region of *TAS1R1* (Fig. 1a–d) or *TAS1R3* (Fig. 1f). The frameshift mutation that is not accompanied by a premature stop codon (Fig. 1e) effectively changes the sequence composition of 36 3′-terminal original codons of *TAS1R1* exon 6 and lengthens this exon. All these mutations indicate that *TAS1R1* and *TAS1R3* of *L. patagonicus* and *TAS1R1* of *G. cuja* are pseudogenes, which predicts that TAS1R1–TAS1R3 is nonfunctional in both species.

**Fig. 2.**
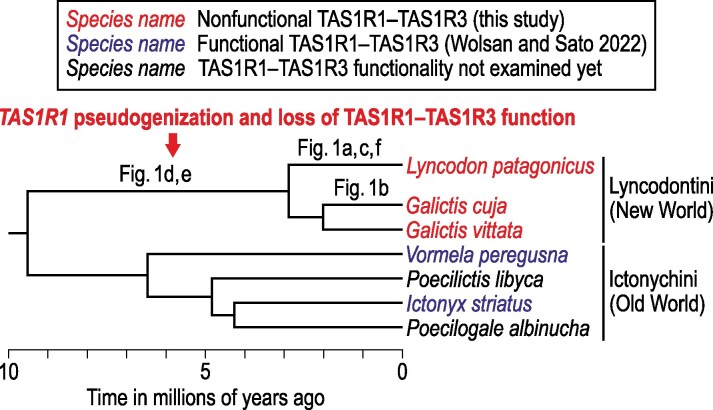
Loss of TAS1R1–TAS1R3 function in the Lyncodontini's stem lineage inferred from mapping of *TAS1R1* and *TAS1R3* inactivating mutations on a time-calibrated phylogeny of Ictonychinae. The mutations are referred to by their figure numbers. The phylogeny is after Sato et al. (2012).

Mapping of all mutations on a dated phylogeny of Ictonychinae (Fig. 2) shows that the frameshift mutations arose between ∼3 and 9.5 million years ago in the stem lineage of Lyncodontini, and that the nonsense mutations arose more recently after division into the *Lyncodon* and *Galictis* lineages. This provides evidence for the loss of TAS1R1–TAS1R3 function in a common ancestor of *Lyncodon* and *Galictis* as a result of *TAS1R1* pseudogenization inflicted by one of the frameshift mutations. This, in turn, predicts that TAS1R1–TAS1R3 is nonfunctional in all species of Lyncodontini, including *G. vittata*, which has not been sampled here. An alternative hypothesis that TAS1R1–TAS1R3 function was lost independently in *L. patagonicus* and *G. cuja* and retained in *G. vittata* is less likely.

We note that the presence of the *TAS1R3* pseudogene in *L. patagonicus*, first reported in this study, confirms our previous prediction (Wolsan and Sato 2022) that this species has a nonfunctional TAS1R2–TAS1R3 sweet taste receptor.

## 4. Discussion

The sense of taste guides vertebrates to consume beneficial nutrients and avoid harmful substances. Although as such it appears crucial for survival and reproduction, evolutionary loss of taste receptor function turns out to be a frequent and widespread phenomenon. This phenomenon has been discovered in various lineages of fishes (e.g. Ishimaru et al. 2005; Hashiguchi et al. 2007; Liu et al. 2017), amphibians (e.g. Shi and Zhang 2006; Dong et al. 2009; Zhong et al. 2021), reptiles (e.g. Li and Zhang 2014; Zhong et al. 2017; Feng and Liang 2018), birds (e.g. Baldwin et al. 2014; Wang and Zhao 2015; Zhao et al. 2015), and mammals (e.g. Go et al. 2005; Zhao et al. 2012; Feng et al. 2014; Liu et al. 2016; Wolsan and Sato 2022). Studies have shown that losses of taste receptor function are nonadaptive and reflect a relaxation of functional constraint (Go et al. 2005; Jiang et al. 2012a; Hu et al. 2017; Tarusawa and Matsumura 2020; Wolsan and Sato 2020). This relaxation has been proposed to be caused by feeding specializations because they restrict diet composition and thereby can reduce or deprive of access to the compounds that a particular taste receptor recognizes (Jiang et al. 2012a; Wolsan and Sato 2022). Although this hypothesis is supported by considerable evidence (e.g. Jiang et al. 2012a; Feng et al. 2014; Hong and Zhao 2014; Liu et al. 2016; Wolsan and Sato 2022), it has been subject of controversy. This controversy has arisen from observed mismatches between feeding habits and the functionality of taste receptors (Zhao and Zhang 2012; Feng and Zhao 2013; Feng and Liang 2018; Zhong et al. 2021; Policarpo et al. 2024). However, such mismatches do not necessarily contradict the hypothesized causal role of feeding specialization in loss of taste receptor function because this loss is achieved by a stochastic process that occurs after a species has switched to a restricted diet; this process continues over evolutionary time and can be decelerated or even stopped by extragustatory functions of taste receptor proteins. Therefore, at any one moment in evolutionary time, one should not expect to see a perfect correspondence between a feeding specialization and loss of taste receptor function (Jiang et al. 2012b; Wolsan and Sato 2022).

**Fig. 3.**
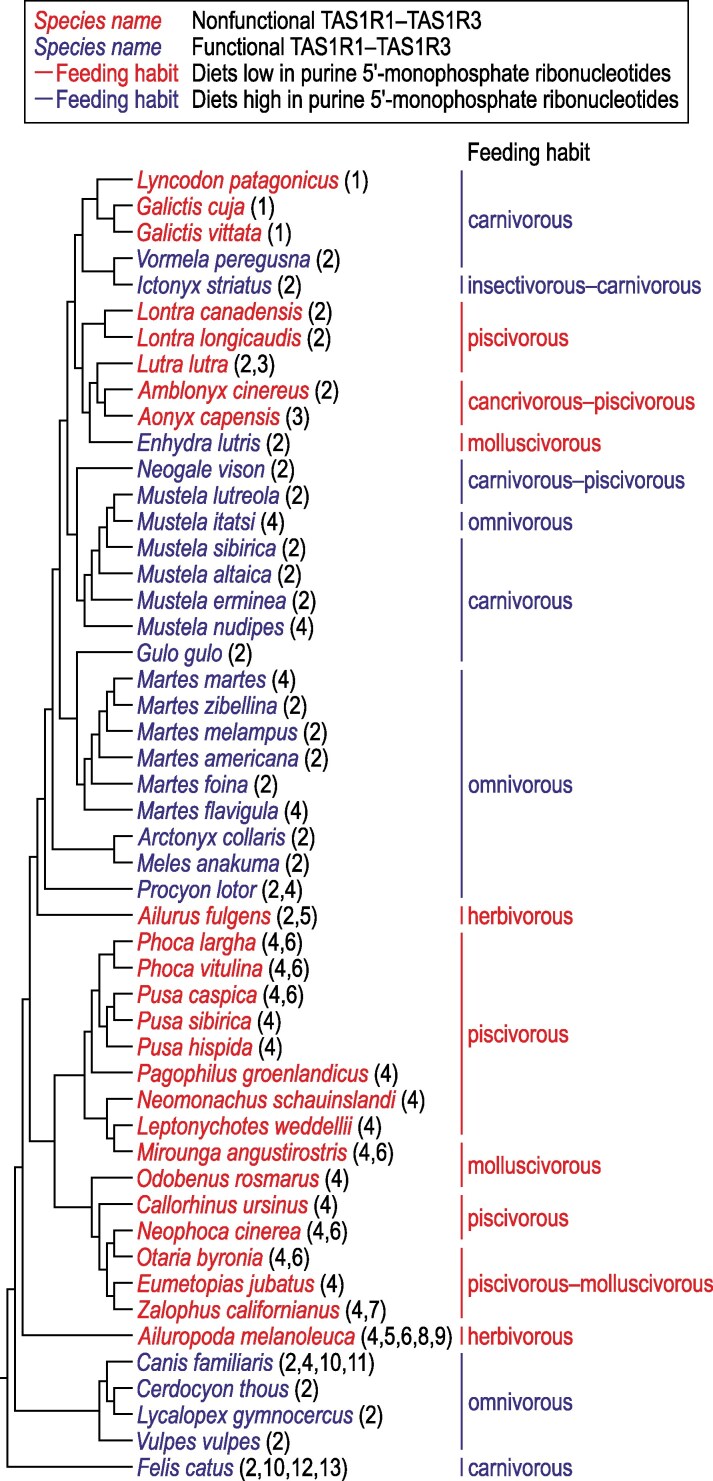
Functional conditions of TAS1R1–TAS1R3 in relation to feeding habits in Carnivora. References for the functional conditions: 1, this study; 2, Wolsan and Sato (2022); 3, Tarusawa and Matsumura (2020); 4, Wolsan and Sato (2020); 5, Hu et al. (2017); 6, Sato and Wolsan (2012); 7, Jiang et al. (2012a); 8, Li et al. (2010); 9, Zhao et al. (2010); 10, Li et al. (2005); 11, Shi and Zhang (2006); 12, Beauchamp et al. (1977); 13, McGrane et al. (2023). Feeding habits are defined in Supplementary Table S5. Species are assigned to the feeding habits based on data from Wilson and Mittermeier (2009, 2014), Pauly et al. (1998), Prevosti et al. (2009), and Yensen and Tarifa (2003a, 2003b). Phylogenetic relationships are compiled from Sato et al. (2012), Wolsan and Sato (2020), Koepfli et al. (2008), Lindblad-Toh et al. (2005), and Eizirik et al. (2010).

A confrontation of available data on functionality of TAS1R1–TAS1R3 with feeding habits in Carnivora (Fig. 3) shows that data derived from previous studies are in agreement with an idea that loss of TAS1R1–TAS1R3 function is caused by feeding specializations that substantially restrict access to purine 5′-monophosphate ribonucleotides, which are the main agonists of TAS1R1–TAS1R3 in carnivorans (Toda et al. 2021a; McGrane et al. 2023). Specifically, all previously examined carnivoran species that include tetrapods in their diets (all carnivores and omnivores, insectivores–carnivores, and carnivores–piscivores in Fig. 3) possess, as expected, a functional TAS1R1–TAS1R3. A nonfunctional TAS1R1–TAS1R3, in turn, has previously been found only in carnivoran species specialized in feeding on fish, aquatic mollusks, both fish and aquatic invertebrates, or plants (all piscivores and herbivores, cancrivores–piscivores, piscivores–molluscivores, and part of molluscivores in Fig. 3). And this is also expected because purine 5′-monophosphate ribonucleotides are relatively scarce in tissues of living or recently dead fish and aquatic invertebrates and in most plants (Arai and Saito 1961; Arai and Terasaki 1966; van Waarde 1988; Yamaguchi and Ninomiya 2000; Kurihara 2009; Kaneko et al. 2014). Note that although purine 5′-monophosphate ribonucleotides accumulate postmortem, they are scarce or absent in living fish and aquatic invertebrates (Arai and Saito 1961; Arai and Terasaki 1966; van Waarde 1988); this is relevant for semiaquatic carnivorans, which hunt in water and mostly feed on alive or recently dead fish and/or aquatic invertebrates.

The only unexpected case reported previously from Carnivora is the retention of a functional TAS1R1–TAS1R3 in *Enhydra lutris* despite the fact that this species is molluscivorous, and thus its diet is predicted to be low in purine 5′-monophosphate ribonucleotides (Fig. 3). However, this case not necessarily contradicts the hypothesis that loss of TAS1R1–TAS1R3 function is caused by feeding specializations because the process of loss of this receptor's function, albeit not yet completed in this species, could have begun as predicted by this hypothesis. This is likely given the facts that this process is stochastic and depends on evolutionary time, and the TAS1R1 and TAS1R3 proteins have, in addition to their gustatory function, nongustatory functions in extraoral tissues, which can potentially decelerate this process (Wolsan and Sato 2022).

It is of note that Policarpo et al. (2024) contended that there is no significant association between feeding habits and *TAS1R1* or *TAS1R3* gene losses in mammals. However, these authors based this assertion on incomplete data (e.g. relevant data reported by Wolsan and Sato [2022] from Carnivora were not considered) and only 3 feeding habit categories of which the category “carnivores” was inappropriately applied to encompass species that feed on tetrapods and those that feed on fish and/or aquatic invertebrates. Specifically, Policarpo et al. (2024) reported, for mammalian *TAS1R1*, 8 gene losses in “carnivores,” 9 in herbivores, and 5 in omnivores; and for mammalian *TAS1R3*, 6 gene losses in “carnivores,” 9 in herbivores, and 6 in omnivores. In turn, Wolsan and Sato (2022) reported, for carnivoran *TAS1R1*, no gene losses in carnivores and omnivores, 2 in herbivores, and 20 in piscivores, cancrivores–piscivores, piscivores–molluscivores, and molluscivores; and for carnivoran *TAS1R3*, no gene losses in carnivores, herbivores, and omnivores, but 16 in piscivores, cancrivores–piscivores, piscivores–molluscivores, and molluscivores.

*L. patagonicus*, *G. cuja*, and *G. vittata* are exceptional among carnivorans in that they possess a nonfunctional TAS1R1–TAS1R3 despite the diets high in umami taste-eliciting compounds, including purine 5′-monophosphate ribonucleotides (Fig. 3). This is unexpected and apparently inconsistent with the hypothesis of causal relationship between feeding specialization and loss of taste receptor function. However, importantly, the results of this study indicate that the lack of TAS1R1–TAS1R3 function in these species is a remnant from their common ancestor that lived within a time interval of ∼3 to 9.5 million years ago as part of the Lyncodontini's stem lineage. The diet of this common ancestor is unknown but could have differed from those of its extant descendants.

Given that (i) the empirical data for non-Lyncodontini carnivorans presented in Fig. 3 are overwhelmingly congruent with the predictions of the hypothesis of causal relationship between feeding specialization and loss of taste receptor function (Jiang et al. 2012a; Wolsan and Sato 2022), and specifically with its subhypothesis that loss of TAS1R1–TAS1R3 function is caused by feeding specializations that greatly reduce access to purine 5′-monophosphate ribonucleotides; (ii) all non-Lyncodontini carnivorans that have been reported to possess a nonfunctional TAS1R1–TAS1R3 are specialized in feeding on fish and/or aquatic invertebrates or plants (Fig. 3); and (iii) to our knowledge, no mustelid, living or extinct, is known to be specialized in feeding on plants, we find it likely that the ancestor of Lyncodontini that first lost TAS1R1–TAS1R3 function predated mainly on fish, aquatic invertebrates, or both. Because this feeding specialization is associated in Carnivora with a semiaquatic feeding habitat (Wilson and Mittermeier 2009, 2014), and because purine 5′-monophosphate ribonucleotides are scarce or absent in living fish and aquatic invertebrates (Arai and Saito 1961; Arai and Terasaki 1966; van Waarde 1988), we hypothesize that the ancestor in question was semiaquatic. Because loss of taste receptor function is achieved by a stochastic process that continues over evolutionary time, our hypothesis effectively suggests a prolonged semiaquatic episode in the evolutionary history of the Lyncodontini's stem lineage.

Although the fossil record of the Lyncodontini's stem lineage is poorly understood with regard to feeding habits and habitats, what is known supports rather than contradicts our hypothesis of a semiaquatic ancestry for Lyncodontini. Specifically, the earliest reported member of this lineage, the 6 to 7.5 million years old *Lutravus* (Jiangzuo et al. 2024), exhibits an otter-like dentition (Furlong 1932; Jiangzuo et al. 2024) suggestive of otter-like feeding habit and habitat. This fossil clue is in harmony with the well-supported sister-group relationship of Ictonychinae to the otter subfamily Lutrinae (Koepfli and Wayne 2003; Fulton and Strobeck 2006; Eizirik et al. 2010; Wolsan and Sato 2010; Sato et al. 2012; and others), which suggests that a semiaquatic lifestyle may have been basal in the Ictonychinae's total clade. It is of note that, although all extant species of Lyncodontini are primarily terrestrial (Yensen and Tarifa 2003a, 2003b; Prevosti et al. 2009), both species of *Galictis* are often found near water (Yensen and Tarifa 2003a, 2003b), *G. cuja* exhibits some aquatic muscular adaptations shared with otters (Ercoli et al. 2015), and *G. vittata* is an excellent swimmer (Yensen and Tarifa 2003b).

The lack of a functional TAS1R1–TAS1R3 in Lyncodontini raises the question about how the carnivorous species of this tribe sense proteins. One possible explanation may be that another receptor is used for umami perception. It might be taste mGluR1 and/or taste mGluR4 or a newly evolved receptor. For example, although birds lost a functional TAS1R2–TAS1R3 sweet taste receptor during evolution, hummingbirds, songbirds, and woodpeckers can sense sweetness through a repurposed TAS1R1–TAS1R3 (Baldwin et al. 2014; Toda et al. 2021b; Cramer et al. 2022). Whether Lyncodontini actually evolved a mechanism to compensate for the loss of TAS1R1–TAS1R3 function is currently unknown and requires further research.

## Supplementary Material

bjaf045_Supplementary_Data

## Data Availability

The DNA sequence data used in this study are available from DDBJ/ENA/GenBank under accession numbers GCA_000002285.2, LC654496, LC727912, LC727945, LC727953, LC727975, and LC887790–LC887793.
