# Supplementary material for: Unexpected loss of TAS1R1–TAS1R3 umami taste receptor function in carnivorous Lyncodontini mustelids
Source: Chem Senses. 2025 Oct 23;50:bjaf045. doi: 10.1093/chemse/bjaf045 (PMC12596590; doi:10.1093/chemse/bjaf045)
Supplement: bjaf045_Supplementary_Data [file bjaf045_supplementary_data.pdf]

Chemical Senses

Supplementary Data

Unexpected loss of TAS1R1–TAS1R3 umami taste receptor function in carnivorous Lyncodontini mustelids

Mieczysław Wolsan[1] and Jun J. Sato[2]

[1]Museum and Institute of Zoology, Polish Academy of Sciences, Twarda 51/55, 00-818 Warszawa, Poland
[2]Department of Biological Science, Fukuyama University, Higashimura-cho, Aza, Sanzo, 985-1, Fukuyama 729-0292, Japan

Contents

Table S1: Origin and accessibility of *TAS1R1* and *TAS1R3* sequence data obtained in this study 1
Table S2: Primer pairs used to amplify *TAS1R1* exons in *Lyncodon patagonicus* 2
Table S3: Primer pairs used to amplify *TAS1R3* exons in *Lyncodon patagonicus* 3
Table S4: Primer pairs used to amplify *TAS1R1* exons in *Galictis cuja* 4
Table S5: Definitions of feeding habits 5
References 5

Table S1. Origin and accessibility of *TAS1R1* and *TAS1R3* sequence data obtained in this study.

Species	Voucher specimen number	DNA sample number	Locality	DDBJ/ENA/GenBank accession numbers	
				TAS1R1	*TAS1R3*
Lyncodon patagonicus	MC 379	JS 266	Puerto Madryn, Chubut, Argentina	LC887790, LC887791	LC887793
Galictis cuja	MC 795	JS 270	42°28'10" S, 64°22'06" W, Valdes Peninsula, Chubut, Argentina	LC887792	—

Abbreviations: JS, Jun Sato's collection deposited in the Department of Biological Science, Fukuyama University, Japan; MC, Marcelo Carrera's collection, Puerto Madryn, Argentina.

Table S2. Primer pairs used to amplify *TAS1R1* exons in *Lyncodon patagonicus.*

Forward primer			Reverse primer		
**Name**	**5′ to 3′ sequence**	**Reference**	**Name**	**5′ to 3′ sequence**	**Reference**
**Exon 1**					
Tas1r1_ex1_MustelaF1	CTCTGCTCGGCCATGCCAGGCG	1	Tas1r1_ex1_MustelaR1	TGGCGCCAGGGAAGGTCTAGCC	1
Tas1r1_ex1_MustelaF2	GAAATTGGGAAGCATCTGGGCGGC	2	Tas1r1_ex1_MustelaR1	TGGCGCCAGGGAAGGTCTAGCC	1
Tas1r1_Ex1_Fw2	GGCCAGCATGTCACTCCTGGCAGCT	2	Tas1r1_Ex1_Rv2	CACCGTGGGCCTGCGTCTCACC	2
T1R1_EX1_F1	GGCCATGCCAGGCACAGGAC	3	Tas1r1_ex1_dogpandaR1	CCCCAGAGGCAGAAAGGGCA	1
T1R1_EX1_F1	GGCCATGCCAGGCACAGGAC	3	T1R1_EX1_R1	CCCCTCACTCACCTGTCACAGAGRGT	3
**Exon 2**					
Tas1r1_ex2_MustelidaeIntF1	GACGTGTGCTCCGAGTCGGCCAA	2	Tas1r1_ex2_MustelaR1	GCAGAACCCCCTGCAATGTGAC	2
T1R1_EX2_F1	GCTCTCAGCYKGGCTTTCTCYACAG	3	T1R1_EX2_R2_2	CACAGGCACCAGAAAGGGGCTCA	1
**Exon 3**					
Tas1r1_ex3_Fw1	GGAGTGAAGCGGTATTACCC	4	Tas1r1_ex3_Rv1	GGCCTCTTCAAACTCCTTCAGGC	4
Tas1r1_ex3_GuMeIcLuF1	TGTGCCTCTGGAGCCTGTTCCAGGG	2	Tas1r1_ex5_MustR2	CTCGCTGGTGCCCTTCAAGACAGT	2
Tas1r1_ex3_MustelaCanidF1	CTGAACTTGCTGAACGGAACCCC	2	Tas1r1_ex3_MusteloidPinniR1	GGCCAGCTGCCTGCTAGAGAA	1
Tas1r1_ex3_MustF1	ATTGGCACGGTGCTGGGTGTGGCCAT	2	Tas1r1_ex3_MusLutGulR1	CAGGTAATCACCCTGTTTCAGGG	1
Tas1r1_ex3_MustF1	ATTGGCACGGTGCTGGGTGTGGCCAT	2	Tas1r1_ex3_MustelidaeR1	GTGAAGCATCATGGCCTGCATC	2
**Exon 4**					
Tas1r1_ex3-4_IctF1	CCTCTCTTAGGCTTCGCTTCTC	This study	Tas1r1_ex4_MustelidaeR1	AGGCTCCAGGCTGCCCATAAGGC	This study
Tas1r1_ex3-4_IctF2	AGAAGCTTCCTCTCAGGAGGC	This study	Tas1r1_ex4_IctR2	TGTCCAGAGCATCCCCAGGCT	This study
Tas1r1_ex3-4_IctF2	AGAAGCTTCCTCTCAGGAGGC	This study	Tas1r1_ex4_MustelidaeR1	AGGCTCCAGGCTGCCCATAAGGC	This study
Tas1r1_ex4_MustCaniF1	GGCCTCCAGTTCAGCTGGACAT	2	Tas1r1_ex5_IctR1	CTCGCCGGTGCCCTTCARGACAGT	2
Tas1r1_ex4_MustCaniF1	GGCCTCCAGTTCAGCTGGACAT	2	Tas1r1_ex5_itatsiR3	CCCCTCATCTGCTCTATAGACCC	2
Tas1r1_ex4_MustIctF1	GAGAAGGAACGGTCTTTGC	2	Tas1r1_ex4_MustIctR1	CCAGGCTCCAGGCTGCCCATAA	2
**Exon 5**					
Tas1r1_ex3_MustF1	ATTGGCACGGTGCTGGGTGTGGCCAT	2	Tas1r1_ex5_MustR2	CTCGCTGGTGCCCTTCAAGACAGT	2
Tas1r1_ex5_MustIctF3	AAAACACCTGTGGCTTCTTGC	This study	Tas1r1_ex5_caniformR2_last	AAGACATAGTGGGTCTGAGGA	2
Tas1r1_ex5_MustIctF4	GCAGGTGCCTAAGTCCGTGTGC	2	Tas1r1_ex5_caniformR1_last	GACCCTAGGAGTGCCCAGTC	2
T1R1_EX5_F1	CAGAACACCTGTGGCTTCTTGCAGGT	3	T1R1_EX5_R1	CACTCACCRCTCTTGTTGAGGAAGSTSC	3
**Exon 6**					
Tas1r1_ex6_intMustF2	GGCCCAGCTGCTCATCTGT	2	Tas1r1_ex6_MustR1	CGCCTCGCTCGACCCGCGAC	2
Tas1r1_ex6_MustLutF2	CCTCACTCCTGTTCTGCTCATC	2	Tas1r1_ex6_intMustR1	CCTTGCCCAGGTAGCTGCAGGCAAAG	2
T1R1_EX6_F1	CCTTTYCTTCCAGACCTCCACAGMTGC	3	T1R1_EX6_intR1	AAAGGCGCTGACGGACAGGA	1
T1R1_EX6_intF1	TACCAGCGCTTCCCTCAGCT	1	T1R1_EX6_R1	TCAGGTGGAGCCGCAGCGCC	3

References: 1, Wolsan and Sato (2020); 2, Wolsan and Sato (2022); 3, Jiang et al. (2012); 4, Sato and Wolsan (2012).

Table S3. Primer pairs used to amplify *TAS1R3* exons in *Lyncodon patagonicus*.

Forward primer			Reverse primer		
**Name**	**5′ to 3′ sequence**	**Reference**	**Name**	**5′ to 3′ sequence**	**Reference**
**Exon 1**					
Tas1r3_ex1_ArctoidF2	GATTTTGCTAAGCAAATCCTCTGC	1	Tas1r3_ex1_MustelidaeR2	TGGGGCCACACAGGGCTGTGGG	1
**Exon 2**					
Tas1r3_ex2_CarniF1onEx1	CCAATGCCACYGTGTGCACCAG	1	Tas1r3_ex2_CarniR1onEx3	TTGCTCAGCCGGTCGGTGCTGGC	1
Tas1r3_ex2_IctF1	TGTTCTTGAGTGGGGACAGGGT	2	Tas1r3_ex2_MustelidaeR2	GTAGCTGACCTGCARGGACAGCG	2
**Exon 3**					
Tas1r3_ex3_intMusLutF2	GCTGGTGCTCTTCTCCTCCGCC	2	Tas1r3_ex3_MustelidaeR1	KGGGGGCTCCCTGCCCTTAC	2
Tas1r3_ex3_MusLutF1	TTCTTCCTCATGCCTCAGGTGTGC	2	Tas1r3_ex3_intArctoidR1	CACCCACACCTTGGGCGAGAGCC	1
**Exon 4**					
Tas1r3_ex4_CaUrMuF1onEx3	GTGTAYGGCGTGGCCCAGGCCCT	2	Tas1r3_ex4_MustelaR1onEx5	CCGGGAGCACTGCGACACAGGC	2
**Exon 5**					
Tas1r3_ex5_MustelaF1	CAGCCAAGCACAACCTGAGCCCC	2	Tas1r3_ex5_CarniR2onEx6	GGTCYGGGGACCACTGGTYCTG	1
**Exon 6**					
Tas1r3_ex6F_CanifF2onEx5	GGCTTCCRCTCCTGCTGTTA	1	Tas1r3_ex6F_intMustR1	AATGTGGAGCAGTGGCTGCTGGG	2
Tas1r3_ex6L_intArctoidF1	GGTGACGGACTGGTGGGTGCTGC	1	Tas1r3_ex6L_ArctoidR2	AGTCARCYTGCAGTCAGGATC	2
Tas1r3_ex6M_intMustelidaeF1	CCACTGGTTCAGGCCTCAGGTGG	2	Tas1r3_ex6M_intMustelidaeR1	GGAAGGCCAGCACAGCATTGG	2

References: 1, Wolsan and Sato (2020); 2, Wolsan and Sato (2022).

Table S4. Primer pairs used to amplify *TAS1R1* exons in *Galictis cuja*.

Forward primer			Reverse primer		
**Name**	**5′ to 3′ sequence**	**Reference**	**Name**	**5′ to 3′ sequence**	**Reference**
**Exon 1**					
Tas1r1_ex1_MustelaF1	CTCTGCTCGGCCATGCCAGGCG	1	Tas1r1_ex1_MustelaR1	TGGCGCCAGGGAAGGTCTAGCC	1
T1R1_EX1_F1	GGCCATGCCAGGCACAGGAC	3	T1R1_EX1_R1	CCCCTCACTCACCTGTCACAGAGRGT	3
**Exon 2**					
Tas1r1_ex2_MustelidaeIntF1	GACGTGTGCTCCGAGTCGGCCAA	2	Tas1r1_ex2_MustelaR1	GCAGAACCCCCTGCAATGTGAC	2
T1R1_EX2_F1	GCTCTCAGCYKGGCTTTCTCYACAG	3	T1R1_EX2_R2_2	CACAGGCACCAGAAAGGGGCTCA	1
**Exon 3**					
Tas1r1_ex3_Fw1	GGAGTGAAGCGGTATTACCC	4	Tas1r1_ex3_Rv1	GGCCTCTTCAAACTCCTTCAGGC	4
Tas1r1_ex3_GuMeIcLuF1	TGTGCCTCTGGAGCCTGTTCCAGGG	2	Tas1r1_ex5_MustR2	CTCGCTGGTGCCCTTCAAGACAGT	2
Tas1r1_ex3_MustF1	ATTGGCACGGTGCTGGGTGTGGCCAT	2	Tas1r1_ex3_MusLutGulR1	CAGGTAATCACCCTGTTTCAGGG	1
Tas1r1_ex3_MustF1	ATTGGCACGGTGCTGGGTGTGGCCAT	2	Tas1r1_ex3_MustelidaeR1	GTGAAGCATCATGGCCTGCATC	2
**Exon 4**					
Tas1r1_ex4_ArctoidF1	GGCAGCTCCCTGATCCCTGTTTA	1	Tas1r1_ex5_IctR1	CTCGCCGGTGCCCTTCARGACAGT	2
Tas1r1_ex4_MustCaniF1	GGCCTCCAGTTCAGCTGGACAT	2	Tas1r1_ex5_IctR1	CTCGCCGGTGCCCTTCARGACAGT	2
T1R1_EX4_F1	TATYTCAGCTTCTRGAGCAGATCCGY	3	T1R1_EX4_R1	TTACCTGGTTGTCCTYYCCGTGCC	3
**Exon 5**					
Tas1r1_ex5_MustIctF3	AAAACACCTGTGGCTTCTTGC	This study	Tas1r1_ex5_caniformR2_last	AAGACATAGTGGGTCTGAGGA	2
Tas1r1_ex5_MustIctF4	GCAGGTGCCTAAGTCCGTGTGC	2	Tas1r1_ex5_caniformR1_last	GACCCTAGGAGTGCCCAGTC	2
T1R1_EX5_F1	CAGAACACCTGTGGCTTCTTGCAGGT	3	T1R1_EX5_R1	CACTCACCRCTCTTGTTGAGGAAGSTSC	3
**Exon 6**					
Tas1r1_ex6_CanidF2	CCTTCCTCATTCCTGAACTGCT	2	T1R1_EX6_intR1	AAAGGCGCTGACGGACAGGA	1
Tas1r1_ex6_intMusteloideaF1	GCTTCATGTTGGCTTTCGCCTAC	2	Tas1r1_ex6_MustelidaeR3_last	CGGAGGAGGTGTCAGGCCCGCC	2
Tas1r1_ex6_MustelidaeF1_last	CACTCCTGTTCTGCTCATCTAGC	2	Tas1r1_ex6_MustelidaeR1_last	GTAGCTGCAGGCAAAGGCGCTGA	2
T1R1_EX6_F1	CCTTTYCTTCCAGACCTCCACAGMTGC	3	T1R1_EX6_intR1	AAAGGCGCTGACGGACAGGA	1
T1R1_EX6_intF1	TACCAGCGCTTCCCTCAGCT	1	T1R1_EX6_R1	TCAGGTGGAGCCGCAGCGCC	3

References: 1, Wolsan and Sato (2020); 2, Wolsan and Sato (2022); 3, Jiang et al. (2012); 4, Sato and Wolsan (2012).

Table S5. Definitions of feeding habits.

Feeding habit	Definition
Cancrivorous–piscivorous	Annual diet consists of >75% crustaceans and fish, with >50% crustaceans
Carnivorous	Annual diet consists of >80% tetrapods
Carnivorous–piscivorous	Annual diet consists of >80% tetrapods and fish, with 55–70% tetrapods and 25–35% fish
Herbivorous	Annual diet consists of >80% bamboo
Insectivorous–carnivorous	Annual diet consists of >85% insects and tetrapods, with 50–65% insects and 30–45% tetrapods
Molluscivorous	Annual diet consists of >75% molluscs
Omnivorous	Annual diet consists of tetrapods, other animals, and plants, with <80% tetrapods, <60% other animals, and <70% plants
Piscivorous	Annual diet consists of ≥60% fish
Piscivorous–molluscivorous	Annual diet consists of >75% fish and molluscs, with 35–55% fish and 35–55% molluscs

All definitions are after Wolsan and Sato (2022).

References

Jiang P, Josue J, Li X, Glaser D, Li W, Brand JG, Margolskee RF, Reed DR, Beauchamp GK. Major taste loss in carnivorous mammals. *Proc Natl Acad Sci USA*. 2012;109(13):4956–4961. https://doi.org/10.1073/pnas.1118360109

Sato JJ, Wolsan M. Loss or major reduction of umami taste sensation in pinnipeds. *Naturwissenschaften*. 2012;99(8):655–659. https://doi.org/10.1007/s00114-012-0939-8

Wolsan M, Sato JJ. Parallel loss of sweet and umami taste receptor function from phocids and otarioids suggests multiple colonizations of the marine realm by pinnipeds. *J Biogeogr*. 2020;47(1):235–249. https://doi.org/10.1111/jbi.13749

Wolsan M, Sato JJ. Role of feeding specialization in taste receptor loss: insights from sweet and umami receptor evolution in Carnivora. *Chem Senses*. 2022;47:bjac033. https://doi.org/10.1093/chemse/bjac033
